# Circular RNA circASPM promotes the progression of glioblastoma by acting as a competing endogenous RNA to regulate miR-130b-3p/E2F1 axis

**DOI:** 10.7150/jca.57691

**Published:** 2022-03-14

**Authors:** Dianqi Hou, Zhenlin Wang, Haimeng Li, Juan Liu, Yaohua Liu, Yang Jiang, Meiqing Lou

**Affiliations:** 1 Department of Neurosurgery, Shanghai General Hospital of Nanjing Medical University, Shanghai 201620, China.; 2 Division of Experimental Neurosurgery, Department of Neurosurgery, Heidelberg University Hospital, Heidelberg, Germany.; 3 Department of Neurosurgery, Shanghai University of Medicine & Health Sciences Affiliated Zhoupu Hospital, 1500 Zhouyuan Rd, Pudong New District, Shanghai, China.; 4 Department of Neurosurgery, Shanghai Tenth People's Hospital, Tongji University School of Medicine, Shanghai 200072, China.

**Keywords:** Glioblastoma, circASPM, Glioma stem cells, miR-130b-3p, E2F1

## Abstract

**Background:** Glioblastoma Multiform (GBM) is the primary malignancy with the highest incidence and worst prognosis in the adult CNS. Circular RNAs (circRNAs) are a novel and widely diverse class of endogenous non-coding RNAs that can promote or inhibit gliomagenesis. Our study aimed to explore the role of circASPM in GBM and its molecular mechanism.

**Methods:** Levels of circASPM, miR-130b-3p and E2F1 were determined by quantitative real-time PCR (qRT-PCR) or western blotting assay. MTS, Edu, neurospheres formation and extreme limiting dilution assays were used to detect the tumorigenesis and proliferation of GSCs *in vitro*. The interactions between miR-130b-3p and circASPM or E2F1 were demonstrated via qPCR, western blotting, dual-luciferase reporter and RNA immunoprecipitation (RIP) assays. Xenograft experiments were used to analyze tumor growth *in vivo*.

**Results:** CircASPM was overexpressed in GBM and promoted the tumorigenesis and proliferation of GSCs both *in vitro* and *in vivo*. Mechanistically, circASPM up-regulated the expression of E2F1 in GSCs via miR-130b-3p sponging. We furtherly demonstrated that circAPSM could promote the GSCs proliferation via E2F1 up-regulating. Therefore, our study identified a novel circRNA and its possible mechanism in the development and tumorigenesis of GBM.

**Conclusions:** CircASPM can promote GBM progression via regulating miR-130b-3p/E2F1 axis, suggesting that circAPSM could provide an effective biomarker for GBM diagnosis and prognostic evaluation and possibly being used for molecular targeted therapy.

## Introduction

Glioma is the primary malignancy with the highest incidence in the adult CNS, accounting for approximately 70% of all primary intracranial tumors 1. Especially Glioblastoma Multiform (GBM) has a median survival of fewer than 15 months 2, 3. The main current treatment options for GBM include surgical resection combined with chemotherapy (temozolomide, etc.) and local radiotherapy 4, 5. Immunotherapy such as monoclonal antibodies and dendritic cell vaccines are also options, but the results are still unsatisfactory. Glioma stem cells (GSCs) exist in GBM, which have tumor initiation, multi-directional differentiation potential and self-renewal ability, leading to tumor cell infiltrative growth, strong aggressiveness and a high likelihood of radiotherapy resistance, so GBM patients are prone to relapse after treatment and are difficult to be cured, ultimately leading to poor prognosis 6, 7. Recent studies have confirmed that the occurrence and development of GBM are closely related to the aberrant expression of various oncogenes or tumor suppressor genes 8, and gene-targeted therapy has been tremendously developed, so identifying the relevant genes in GBM that promote the malignant phenotype of GSCs will help to reveal the biological mechanisms of GBM, refine therapeutic strategies, and improve patient prognosis.

With the development of sequencing technology, it has been found that coding genes account for less than 10% of the human genome, while non-coding genes are the majority, and these transcripts are called non-coding RNAs (ncRNAs) 9. Circular RNAs (circRNAs) are a novel and widely diverse class of endogenous ncRNAs, thousands of which exist in mammalian cells, which, unlike linear RNAs, form themselves into a covalently closed continuous loop with no 5' or 3' polarity 10, 11. CircRNAs have multiple pathways to exert biological effects, such as mediating transcription, interacting with RNA-binding proteins, acting as competitive endogenous RNAs (ceRNAs) 12-14, or play the role of microRNAs (miRNAs) sponges to regulate transcription and modify parental gene expression 11. Dysregulation of circRNAs expression in cancer can promote or inhibit cancer proliferation, tumorigenesis, apoptosis, and angiogenesis 15. Recent studies have confirmed that multiple circRNAs play significant roles in the course of GBM. For example, circ-0014359 promotes GBM progression by targeting the miR-153/PI3K signaling pathway 16, and circHIPK3 regulates the developmental malignant phenotype of GBM by targeting miR-654 from IGF2BP3 17. Our previous study focused on the role of ASPM in glioma, and several studies have reported the promoting effect of ASPM in gliomas 18. Since circular RNA has become a hot spot in cancer research, we focused on the circular form of ASPM and found that circASPM (circBase ID: hsa_circ_0015772, chr1:197099044-197113230) was obviously overexpressed in GBM tissues with lower patient survival through bioinformatics analysis and clinical specimen detection. However, the cancer-promoting function of circASPM has not yet been reported in the academic community.

MicroRNAs are a highly conserved group of small endogenous non-coding RNAs (19-25 nucleotides in length) in eukaryotes 19. It plays a key role in regulating proliferation, differentiation and apoptosis by binding to the 3'-untranslated regions of downstream mRNAs 20. Existing studies have demonstrated that aberrant miRNAs expression is associated with the initiation and development of various types of tumors 21, 22, and it also contributes to the course of GBM 23. Alterations in miRNA expression can positively or negatively regulate tumor progression 24. A series of miRNAs have also been used as potential and important diagnose biomarkers in various biological processes and prognosis predicting in GBM 25. MiR-130b-3P can act as a tumor suppressor in many tumors, such as nephroblastoma and breast cancer 26, 27. MiR-130b-3p is up-regulated in GBM and inhibits cell proliferation, invasion and migration 28.

E2F transcription factor 1 (E2F1) is the first identified member of the E2F transcription factors family 29. It is involved in the occurrence, metastasis and chemoresistance of several cancers and related to the poor survival time 30. E2F1 could be released after cyclin-dependent kinases (CDKs) phosphorylating pRB, promoting the DNA synthesis, S-phase entry and mitosis in cell cycle progression 31, 32. In addition, it was reported E2F1 mediates circSEPT9 to promote the occurrence and development of triple-negative breast cancer through the circSEPT9/miR-637/LIF axis 33. In glioma, E2F1 could be inhibited by miR-329 to down-regulated cell proliferation by Akt pathway 34.

Our study revealed that circASPM is overexpressed in GBM and can up-regulate E2F1 via miR-130b-3 sponging through bioinformatics analysis. Cellular experiments based on patient-derived GSCs and intracranial tumorigenesis experiments confirmed circASPM could promote GBM initiation, proliferation and the malignant phenotype. Thus, this study identifies circASPM as a novel circRNA associated with the promotion of GBM disease progression and provides a new therapeutic strategy and theoretical basis for the treatment of GBM.

## Materials and methods

### Patient samples and ethics

We collected 55 clinical samples of glioma patients between January 2010 to December 2014 at the Shanghai General Hospital. 15 samples of grade II, 20 samples of grade III, and 20 samples of grade IV glioma (glioblastoma) were included. There are two neuropathologists who confirmed the histological diagnosis according to the World Health Organization (WHO) classification guidelines. During the same period, ten acute brain injury patient samples were collected as a control group. Clinical information for these samples is outlined in Table S1. This study was approved by the Ethics Committee of the Shanghai General Hospital, and written informed consent was obtained from each patient. All animal experiments were conducted under the supervision of the Animal Ethics Committee of Shanghai Jiao Tong University School of Medicine.

### Cell treatment and GSCs isolation

Patient-derived GSCs were isolated, and neurosphere cultures were performed as previously reported 35. In brief, fresh clinical glioblastoma specimens were dissociated into single cells and cultured in Dulbecco's modified Eagle's medium (DMEM) with B27 (1:50), recombinant human (rh) basic fibroblast growth factor (20 ng/mL), and rh-epidermal growth factor (20 ng/mL, Gibco, Gaithersburg, MD, USA). Neurospheres were then collected and routinely cultured in the above-mentioned neurosphere medium. The cancer stem cell nature of isolated GSCs was confirmed by self-renewal and functional assays of tumor formation *in vivo*. The expression of stem cell markers (CD133 and nestin+) and the multi-lineage differentiation capacity of GSCs were detected by immunofluorescence. The detailed clinicopathological information is presented in Supplementary Table 2.

### Lentiviral vector construction and transfection

The artificial repeats and whole sequence of circASPM and E2F1 were subcloned into pcDNA3.1 circRNA mini vector and pcDNA3.1 vector. GenePharma (Shanghai, China) engineered siRNA sequences targeted to circASPM and E2F1 to silence them: circASPM-KD1: sense: 5′-AAUGGUAUCCCAAAGACUCCU-3′, antisense: 5′-GAGUCUUUGGGAUACCAUUAA-3′, circASPM-KD2: sense: 5′-UCUUCUUUUUAAUGGUAUCCC-3′, antisense: 5′-GAUACCAUUAAAAAGAAGAAA-3′. E2F1-KD: sense: 5′-AAAUAACGCUCCAUUAAAGCU-3′, antisense: 5′-CUUUAAUGGAGCGUUAUUUAU-3′. The miR-130b-3p mimic, inhibitor, and their negative controls were obtained from Thermo Fisher Scientific (Assay ID: MC23984 and MH23984; Thermo Fisher Scientific, Waltham, MA, USA). All cells were examined for resistance to puromycin (Sigma, Santa Clara, CA, USA) for 15 days at a 10 μg/ml concentration after transfection. Mimics and inhibitors of miR-130b-3p were also purchased from GenePharma.

### Quantitative real‐time PCR (qRT‐PCR)

TRIzol Reagent (Invitrogen, Carlsbad, CA, USA) was used for RNA extraction. Use M-MLV reverse transcriptase (Invitrogen) or miScript reverse transcription kit (QIAGEN, Hilden, Germany) to synthesize complementary DNA with 1μgRNA as a template. The recombinant circASPM was synthesized with the technological support from Genewiz and used for absolute quantification PCR. The relative expression of circASPM, ASPM mRNA, miR-130b-3p and E2F1 was calculated through 2^-∆∆Ct^ method. Glyceraldehyde-3-phosphate dehydrogenase (β-actin; for circASPM, ASPM mRNA and E2F1) and U6 (for miR-130b-3p) were used as an internal reference. The sequences of the PCR primer pairs were as follows: circASPM, forward 5′-TCGCCTACTTTGGAATCCTG-3′ and reverse 5′-GGCCTTCTTTGAGTGGTGTC-3′; E2F1, forward 5′-ACGCTATGAGACCTCACTGAA-3′ and reverse 5′-TCCTGGGTCAACCCCTCAAG-3′.

### RNase R treatment

To determine the stability of circASPM, 10μg RNA extracted from the patient-derived cell lines was incubated with RNase R (4U/μg; Epicentre Biotechnologies, Madison, WI, USA) or not for 1 hour at 37°C. Then qRT-PCR was used to detect the relative expression of circASPM and ASPM mRNA.

### Western blotting

Western blotting was performed as described previously 36. The protein samples were prepared by a total cell protein extraction kit (Vazyme, Nanjing, China). BCA protein quantification kit (Vazyme) was used for concentration determination. 40 μg protein samples were transferred to polyvinylidene fluoride membranes (Thermo Fisher Scientific) after sodium dodecyl sulfate-polyacrylamide gel electrophoresis and then blocked with fat-free milk. The primary antibodies against E2F1 (1:1000, #ab112580, Abcam, Cambridge, UK), β-actin (1:2000, #66009-1-Ig, ProteinTech, Chicago, IL, USA) were used. ECL kits (Beyotime Biotechnology, Beijing, China) and IMAGE J software (National Institutes of Health, Bethesda, MD, USA) were used to quantify the signal intensity.

### Immunofluorescence

Immunofluorescence was performed as described previously 37. Cells were fixed, permeabilized and blocked with 4% paraformaldehyde, 0.5% Triton X-100, and 5% BSA, incubated with primary antibody overnight at 4°C, followed with FITC- or rhodamine-conjugated secondary antibody. The antibodies against CD133 (1:1000, #ab222782, Abcam), nestin+ (1:500, #ab18102, Abcam), GFAP (1:1000, #ab4674, Abcam) and β III tubulin (1:1000, #ab7751, Abcam) were used. DAPI (Sigma, Santa Clara, CA, USA) is acquired to stain cell nuclei. A laser scanning confocal microscope (IX71, Olympus, Tokyo, Japan) was used to photograph the expression of immunofluorescence.

### MTS proliferation assay

According to the manufacturer's protocol, the CellTiter 96 Aqueous non-radioactive cell proliferation detection kit (Promega, Madison, WI, USA) was used to evaluate GCS proliferation. An ultraviolet spectrophotometer (Thermo Fisher Scientific) was used to measure the absorption at 495 nm.

### 5-Ethynyl-2'-deoxyuridine (EdU) proliferation assay

EdU assays were performed as previously described^38^. In brief, cell lines were seeded in 24-well plates at a density of 1 × 10^5^ cells per well for 24 h before being added with EdU reagent (Beyotime Biotechnology, Beijing, China) for 2 hours at 37 °C. The cells were fixed with paraformaldehyde, and the percentage of EdU positive cells was calculated by a laser scanning confocal microscope (Olympus).

### Limiting Dilution Neurosphere Formation Assay

The self-renewal ability of GSCs was assessed by the neurosphere formation assay as reported previously 35. Briefly, GSCs were dissociated and seeded into 24-well plates and incubated in a fresh medium for seven days. The relative size and number were counted by an optical microscope (Olympus). *In vitro* limiting dilution method, GSCs were seeded at a gradient of 1, 10, 20, 30, 40 or 50 cells per well into 96-well plates for seven days. Later, the number of neurospheres in each well was analyzed and calculated as previously described 39.

### Luciferase activity analysis

Luciferase reporter assays were performed as previously described 40. Briefly, wild-type circASPM, mutant circASPM, wild-type E2F1 and mutant E2F1 were cloned into the empty pmiRGLO luciferase reporter vector (Promega). Then the Dual-Luciferase Reporter Assay System (Promega) was used to detect luciferase activity according to the manufacturer's protocol. Each experiment was independently repeated three times.

### RNA immunoprecipitation (RIP) assay

RIP assay was performed with the EZ-Magna RIP RNA-binding Protein Immunoprecipitation Kit (Millipore, Darmstadt, Germany). GSCs in different conditions were lysed with RIP lysis buffer and magnetic beads conjunct with antibodies against Ago2 and antibodies against IgG, which was used as a negative control. The immunoprecipitated RNAs were isolated after incubation with proteinase K. Finally, The expressions of circASPM, miR-130b-3p and E2F1 were calculated through qRT-PCR.

### RNA pull down assay

To pull down miRNA by circRNA, biotinylated circASPM probe was synthesized by Cloud-Seq Biotech (Shanghai, China), and oligo probe was referred as a control. To pull down circRNA by miRNA, biotinylated miR-130-3p were also synthesized by Cloud-Seq Biotech. After incubation with magnetic beads for 2hs, cell lysates were incubated with the probes overnight. Then the bound RNAs were washed, purified and detected by qRT-PCR.

### Xenograft experiments

Transfected GSCs were injected (5 × 10^4^ cells per mice) orthotopically into the brains of 6-week-old female BALB/c nude mice (Beijing Vital River Laboratory Animal Technology, Beijing, China). The injection point was 2 mm lateral and 2 mm anterior to the bregma, determined with a stereotaxic apparatus. We observed mice daily for neurological symptoms or death, and the tumor volume was measured according to the formula: V = (D × d^2^)/2, that D was the longest diameter and d was the shortest diameter of the tumor. When neurological symptoms were observed, mice were sacrificed by cervical spine dislocation, and the brains were collected for analysis as previously reported 40. All animal experiments were performed according to the guidelines of the Animal Care Committee of Shanghai Jiao Tong University School of Medicine.

### Immunohistochemistry (IHC)

IHC of mice xenograft tumor specimens was performed as previously described 37. In short, tissue samples embedded in paraffin were cut into 4 µm sections and labeled with a primary antibody against E2F1, ki-67. The sections were imaged using an optical microscope (Olympus), and the staining intensity was evaluated according to the German immunohistochemical scoring system 41.

### Bioinformatic analyses

The data of circRNA expression in gliomas was obtained from Gene Expression Omnibus (GEO) datasets GSE109569. Starbase (starbase.sysu.edu.cn) was used to predict the binding sites between miR-130b-3p and circASPM or E2F1.

### Statistical analysis

SPSS 22.0 software (IBM, Armonk, NY, USA) was used for the statistical analysis of this study. All experiments were repeated more than three times, and the results were expressed as mean ± standard error. The statistical significance between groups was tested by the chi-square test and t-test. The Log-rank test and Kaplan-Meier analysis were used to analyze differences in survival rates. All statistical tests were two-sided tests, and statistical significance is defined as P value < 0.05.

## Results

### CircASPM is up-regulated in glioblastomas tissues and correlated with the progression and poor prognosis

**Figure 1:**
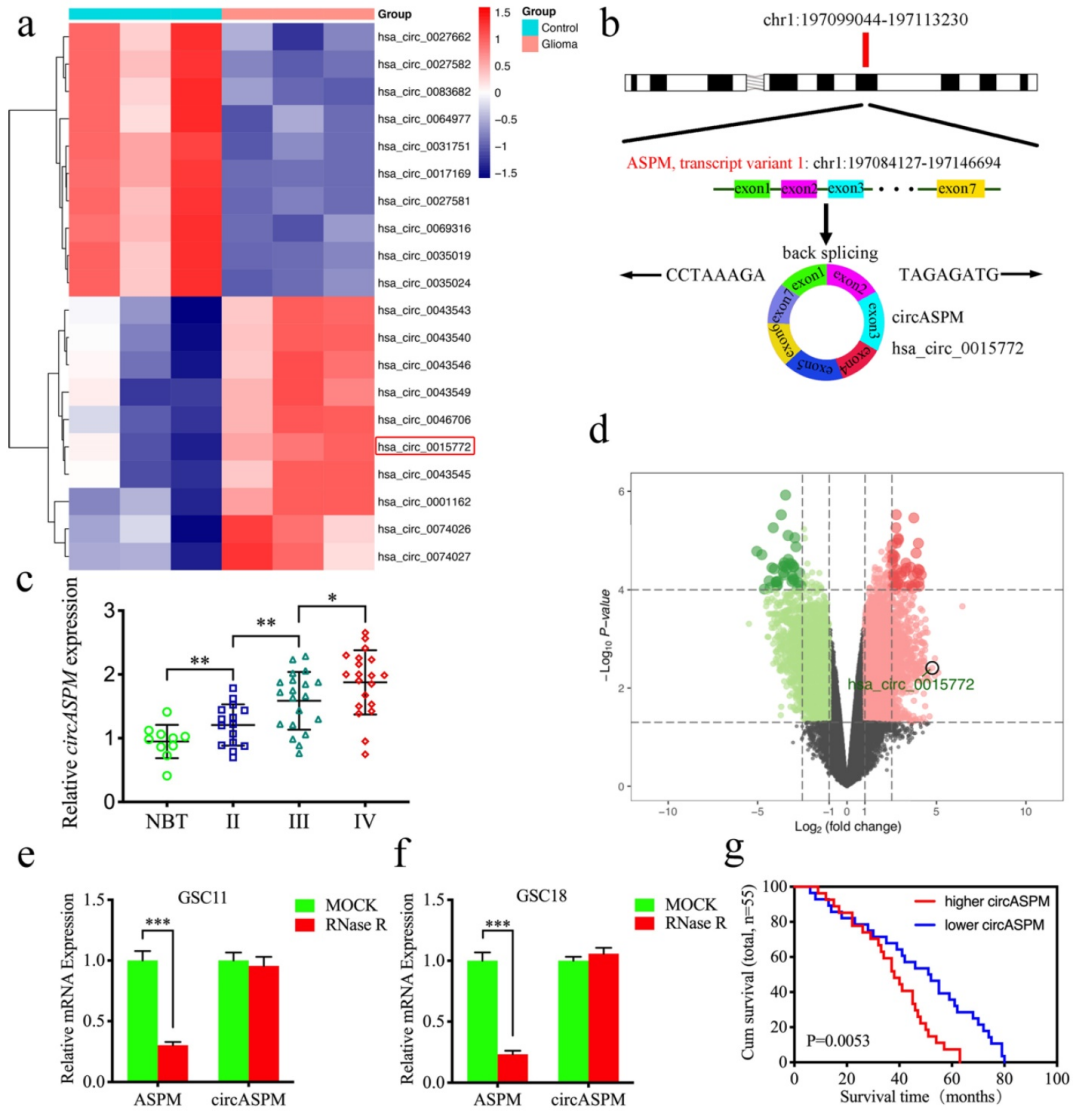
** CircASPM is up-regulated in glioblastomas tissues and correlated with the progression and poor prognosis.** a: Loop formation procedure of circASPM was illustrated. b, c: CircASPM expression was significantly higher in glioma tissues than in normal brain tissues based on GEO datasets GSE109569. d: CircASPM expression was correlated with WHO grades in glioma. e, f: Kaplan-Meier analysis of all patients or GBM patients in terms of higher circASPM expression versus low circASPM expression. g: Sanger sequencing validated the specific junction of circASPM. h, i: qPCR analysis of ASPM mRNA and circASPM in GSC11 and GSC18 treated with Rnase R. j: half-life detection confirmed the circular characteristics of circASPM. All data are shown as the mean ± SD (three independent experiments). *P < 0.05; **P < 0.01; ***P < 0.001.

Hsa_circ_0015772 is derived from transcript 1 of the ASPM gene (chr1:197099044-197113230) and is formed into a loop by reverse splicing its exons 1-7 (Fig. 1a). We first found that hsa_circ_0015772 is significantly higher expression in glioma tissues than in normal tissues based on Gene Expression Omnibus (GEO) datasets GSE109569 (Supplementary Table 3, Fig. 1b, c). To validate the conclusions of bioinformatics analysis, we selected clinical specimens from 55 glioma patients for qPCR assays and confirmed that the copy number of circASPM was up-regulated with increasing WHO grade (Fig. 1d). Subsequently, we performed Kaplan-Meier survival analysis on the prognostic significance of circASPM expression in 55 patients. The results showed that the average survival time of all patients or GBM patients with high circASPM expression was significantly shorter than those with low expression (qPCR quantification, Cutoff: median, Fig. 1e, f). The clinical information of the above 55 samples is outlined in Supplementary Table 1.

We then verified the circularization characteristic and stability of circASPM. Sanger sequencing was conducted to validate the specific junction of circASPM (Fig. 1g). RNase R assays are commonly used to confirm RNAs' circular structure because it can degrade linear RNAs with short 3′-tails, whereas it cannot degrade circRNAs 42. After RNase R disposition, ASPM mRNA expression was significantly decreased in GSC11 and GSC18, while circASPM was unaffected (Fig. 1h, i), indicating that circASPM is more resistant to RNase R digestion. Moreover, the RNA stability assay showed the half-life of circASPM was obviously longer than ASPM (Fig. 1j).

We further extracted patient-derived GSCs from fresh clinical GBM specimens for culture, from which we selected six GSCs in the best growth state for subsequent studies. Supplementary Table 2 shows that the clinical information about the patients from whom the six GSCs were derived. In Supplementary Figure 1a, we show hematoxylin and eosin (H&E) staining of the original patient tumors. Supplementary Figure 1b demonstrated the multi-lineage differentiation capacity of GSCs. Immunofluorescence staining was performed to confirm the expression of the stem cell markers CD133+ and Nestin+ in the isolated neurospheres (Sup. Fig. 1c). Subsequently, we applied qPCR to determine the expression of circASPM in six GSCs and normal human astrocyte (NHA). The copy number of circASPM was obviously higher in GSCs than NHA, especially highest in GSC18 (Sup. Fig. 1d). In summary, circASPM is a stable circRNA that is potentially involved in promoting glioblastomas' malignant phenotype.

### Overexpression of circASPM promoted the proliferation of GSCs *in vitro*

**Figure 2:**
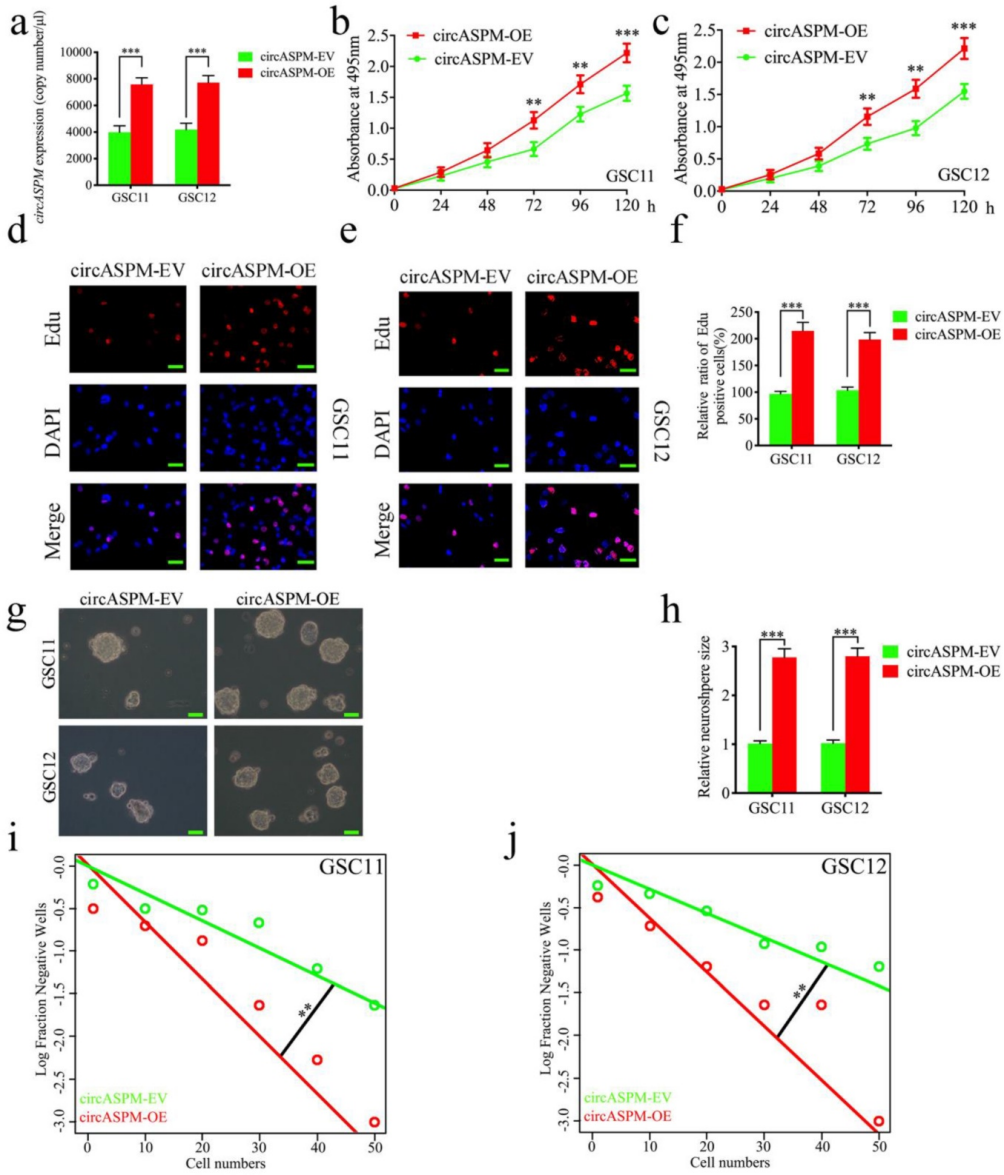
** Overexpression of circASPM promoted the proliferation of GSCs *in vitro*.** a, b: The expression of circASPM in GSC11 and GSC12 after transfection of the circASPM overexpression plasmids as measured by qPCR. c, f: circASPM overexpression significantly increased the proliferation of GSC11 and GSC12 in MTS assays. d, e, f: Proliferative capacity of tumor cells was inhibited after circASPM overexpression as measured by Edu assays. Scale bar = 50μm. g, h, i, j: Representative images of neurospheres and extreme limiting dilution assays showed tumor formation rate up-regulated after circASPM overexpression in GSC11 and GSC12. Scale bar = 50μm. All data are shown as the mean ± SD (three independent experiments). *P < 0.05; **P < 0.01; ***P < 0.001.

We performed a series of gain-of-function assays to demonstrate the role of circASPM in GSCs. We designed lentiviral-based circASPM overexpression plasmids to infect GSC11 and GSC12 and qPCR confirmed that circASPM overexpression was optimal (Fig. 2a), and did not affect the ASPM RNA expression level (Sup. Fig. 1e). First, MTS assays showed the absorbance values of GSCs after circASPM overexpression were significantly higher than those of controls (Fig. 2b, c), confirming that circASPM overexpression promoted the viability of GSCs. Next, we used Edu assays to examine the role of circASPM overexpression on tumor proliferative capacity. The proportion of Edu-positive cells in circASPM overexpressed cell lines were higher than that in controls, indicating that tumor cells' proliferative capacity was promoted after circASPM overexpression (Fig. 2d-f). Furthermore, neurosphere formation assays showed the relative size of spheres and the numbers of spheres after circASPM overexpression were greater than those in the control group (Fig. 2g, h). Extreme limiting dilution assay also showed that circASPM overexpression increased the rate of GSCs formation (Fig. 2i, j). Similar results were also obtained after circASPM overexpression in glioma cell line U87 (Sup. Fig. 2 a-d). The above results showed that circASPM overexpression could promote the proliferation of glioma stem cells.

### Knockdown of circASPM suppressed the proliferation of GSCs *in vitro*

**Figure 3:**
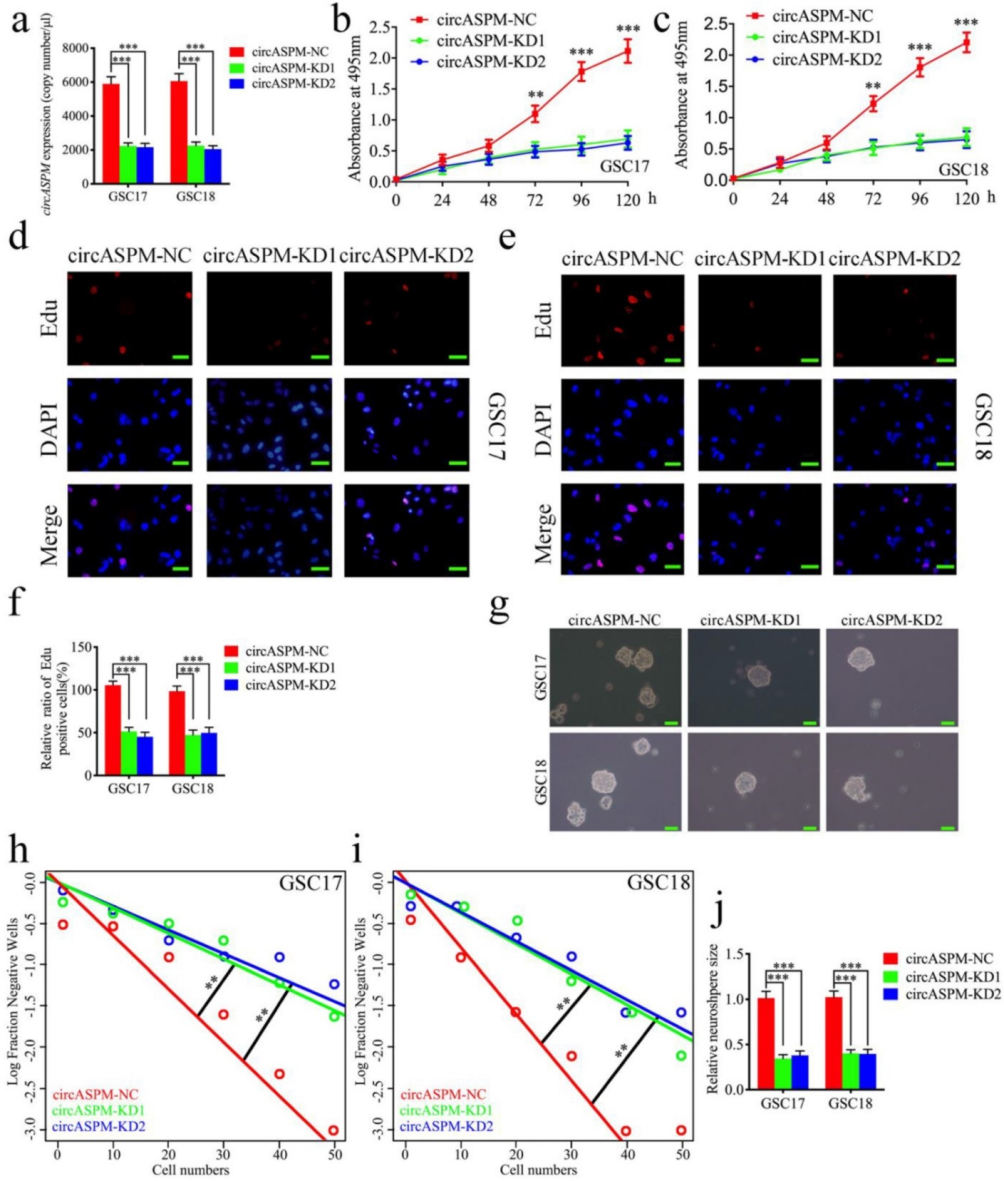
** Knockdown of circASPM suppressed the proliferation of GSCs *in vitro***. a: The expression of circASPM in GSC17 and GSC18 after transfection of circASPM-KD1, circASPM-KD2 or negative control as measured by qPCR. b, c: CircASPM knockdown significantly reduced the proliferation of GSC17 and GSC18 in MTS assays. d, e, f: CircASPM knockdown can significantly increase the proliferative capacity of GSC17 and GSC18 as measured by Edu assays. Scale bar = 50μm. g, j, h, i: Neurospheres formation assays and extreme limiting dilution assays showed that the tumor formation rates decreased after circASPM knockdown in GSC17 and GSC18. Scale bar = 50μm. All data are shown as the mean ± SD (three independent experiments). *P < 0.05; **P < 0.01; ***P < 0.001.

To furtherly demonstrate the function of circASPM on the malignant phenotype of GSCs, we knocked down circASPM by transfecting si-circASPM into GSCs and qPCR was used to confirm the efficiency of circASPM knockdown (Fig. 3a). MTS assays find a significant decrease in the absorbance values after circASPM knockdown, demonstrating that tumor viability was significantly inhibited (Fig. 3b, c). Subsequently, Edu assays showed that knockdown of circASPM significantly down-regulated the proportion of Edu-positive cells (Fig. 3d-f). Besides, the proliferative activity of neurospheres after circASPM knockdown was found to be significantly inhibited in neurosphere formation assays (Fig. 3g, j). The extreme limiting dilution assays also showed a significant decrease in tumor formation after circASPM knockdown compared to the control group (Fig. 3h, i). The above results indicate that knockdown of circASPM can suppress the malignant phenotype of GSCs.

### MiR-130b-3p could bind with circAPSM and mediated the function of GBM cells

**Figure 4:**
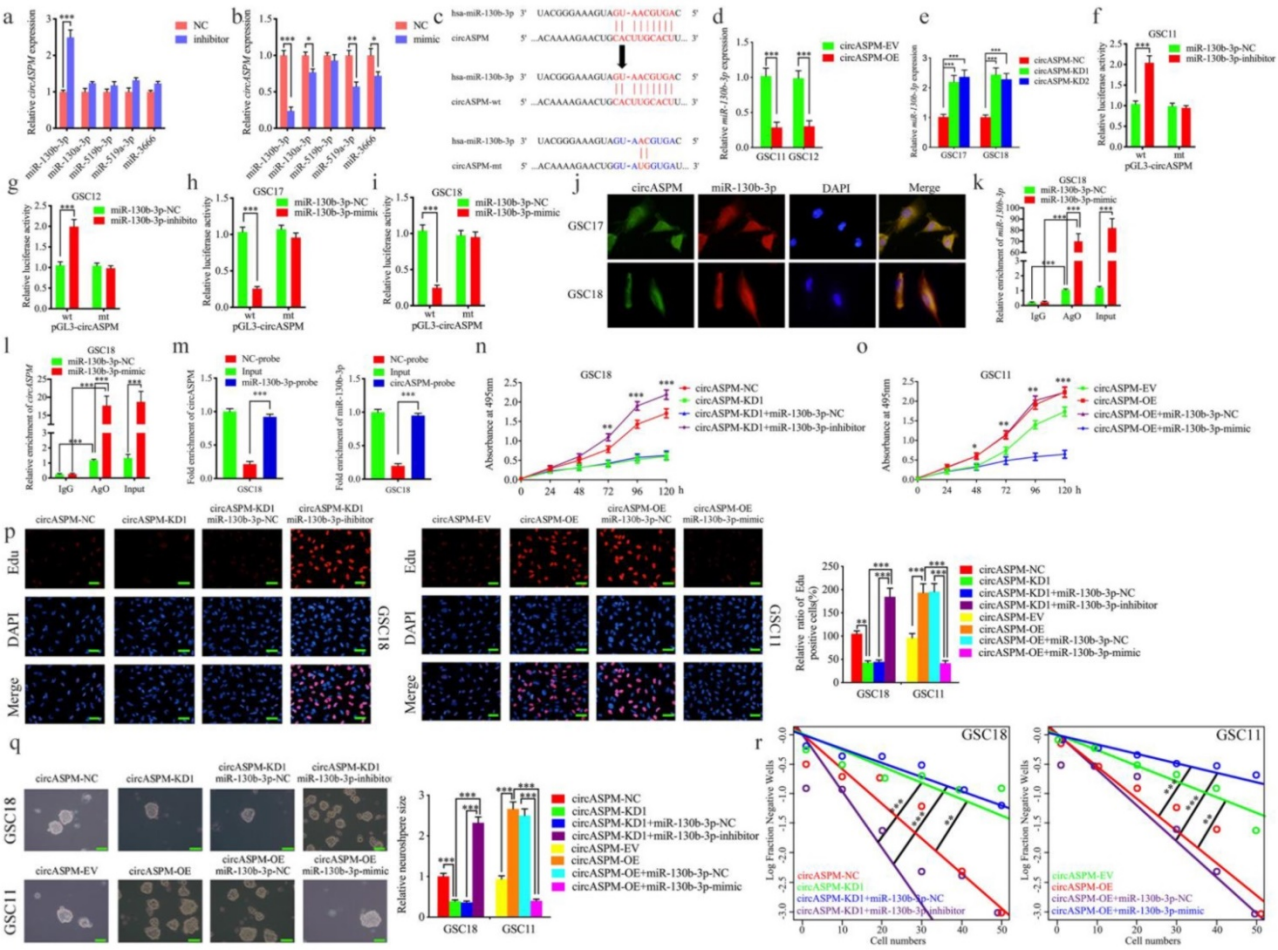
** MiR-130b-3p could bind with circAPSM and mediated the function of GBM cells.** a, b: MiR-130b-3p inhibitor treatment up-regulated the expression of circAPSM while mimic treatment down-regulated circASPM expression as measured by qPCR. c: The predicted binding site between circASPM and miR-130b-3p. d, e: The expression of miR-130b-3p was down-regulated after circAPSM overexpression while circASPM knockdown up-regulated miR-130b-3p expression as measured by qPCR. f, g, h, i: The luciferase reporter assays showed that miR-130b-3p mimic or inhibitor altered the luciferase promoter activities of circASPM. j: FISH assays detected the subcellular localization of circASPM and miR-130b-3p. k, l: CircASPM and miR-130b-3p were effectively pulled down by anti-AGO2 antibodies compared to IgG, and both enriched after miR-130b-3p mimic treatment in GSC18. m: The RNA pull-down assays demonstrated circASPM can sponge miR-130b-3p. n, o: MTS assays showed that circASPM transfection of overexpression plasmids or si-circASPM affected GSCs viability and was reversed by miR-130b-3p mimic or inhibitor treatment, respectively. p: The EDU assay showed that circASPM transfection of overexpression plasmids or si-circASPM affected GSCs proliferation capacity and was reversed by miR-130b-3p mimic or inhibitor treatment, respectively. Scale bar = 50 μm. q, r: In the neurosphere formation assays and extreme limiting dilution assays, circASPM transfection of overexpression plasmids or si-circASPM affected neurosphere growth capacity in GSCs and was reversed by miR-130b-3p mimic or inhibitor treatment, respectively. Scale bar = 50 μm. All data are shown as the mean ± SD (three independent experiments). *P < 0.05; **P < 0.01; ***P < 0.001.

Available studies suggest circRNAs have many microRNA response elements (MREs), and usually regulate the expression of targeted genes through miRNA sponging (2). We first screened out the top 10 miRNAs with the highest AgoExpNum score according to Starbase (Supplementary Table 4). They are hsa-miR-4295, hsa-miR-3666, hsa-miR-301a-3p, hsa-miR-130a-3p, hsa-miR-130b-3p, hsa-miR-301b-3p, hsa-miR-454-3p, hsa-miR-519c-3p, hsa-miR-519b-3p, hsa-miR-519a-3p. Five of them were reported as tumor suppressors (hsa-miR-3666, hsa-miR-130a-3p, hsa-miR-130b-3p, hsa-miR-519b-3p and hsa-miR-519a-3p). Therefore, mimic and inhibitor of these 5 miRNAs were treated and qPCR assays were performed to detect the expression of circASPM. The results showed that miR-130b-3p has the strongest regulation effects (Fig. 4a, b). Figure 4c showed the binding site for miR-130b-3p to circAPSM. We also detected the expression of miR-130b-3p in glioma tissues and GSCs. The results showed miR-130b-3p expressed higher in both NBT and NHA than glioma tissues and GSCs resprespectively (Sup. Fig. 3 a, c). Further, we examined the relative expression of miR-130b-3p by qPCR after overexpression and knockdown of circASPM. MiR-130b-3p expression was also negatively regulated by circASPM levels (Fig. 4d, e).

We then utilized luciferase reporter assays and found increased luciferase activity of wild-type circASPM in GSC11 and GSC12 after miR-130b-3p inhibitor treatment (Fig. 4f, g). In contrast, miR-130b-3p mimic treatment reduced the luciferase activity of wild-type circASPM luciferase in GSC17 and GSC18 (Fig. 4h, i). The similar results were also obtained in glioma cell line U87 (Sup. Fig. 2 e-h). Then, the fluorescence *in situ* hybridization assay has been performed to detect the subcellular localization of circASPM and miR-130b-3p. The results showed both of them were located at cytoplasm (Fig. 4j). Since miRNAs bind to MREs via the RNA-induced silencing complex (RISC), and the Ago 2 (AGO2) protein is a key component of RISC 43, we performed anti-AGO2 RIP assays to determine whether miR-130b-3p and circAPSM are co-enriched in RISC. The results showed anti-AGO2 antibodies effectively pulled down both circASPM and miR-130b-3p compared to IgG. In addition, both circASPM and miR-130b-3p also showed significant enrichment after miR-130b-3p mimic treatment compared to miR-130b-3p negative control (Fig. 4k, l). Furtherly, we performed the RNA pull-down assays and the result showed circASPM was pulled down by biotinylated‐miR‐130‐3p and miR‐130‐3p can aslo be pulled down by biotinylated‐circASPM (Fig. 4m).

We then performed a series of experiments to verify that miR-130b-3p could mediate the malignant phenotype of GBM via binding with circAPSM. MTS assays revealed that circAPSM knockdown in GSC18 could inhibit its proliferation. However, miR-130b-3p inhibitor treatment resulted in stronger tumor proliferation. In contrast, the pro-tumorigenic effect produced by circAPSM overexpression completely disappeared after miR-130b-3p mimic treatment in GSC11, and inhibition even occurred compared to the control (Fig. 4n, o). In Edu assays, the proportion of Edu-positive cells that decreased due to circASPM knockdown increased substantially after miR-130b-3p inhibitor treatment. In contrast, the increase in the proportion of Edu-positive cells due to circASPM overexpression decreased significantly after miR-130b-3p mimic treatment (Fig. 4p). Neurosphere formation assays similarly revealed that circASPM knockdown resulted in relatively smaller size and fewer numbers of GSC18, while miR-130b-3p inhibitor treatment can obviously promoted the neurospheres formation abilities. In contrast, circASPM overexpression induced more and larger neurospheres of GSC11. While After treatment with miR-130b-3p mimic, the size and number of neurospheres were reduced to be even smaller than the control group (Fig. 4q). Extreme limiting dilution assays also showed a similar trend (Fig. 4r). These data suggest that, miR-130b-3p could bind with circAPSM and mediate its function on GBM.

### CircAPSM regulated E2F1 expression via miR-130b-3p sponging

**Figure 5:**
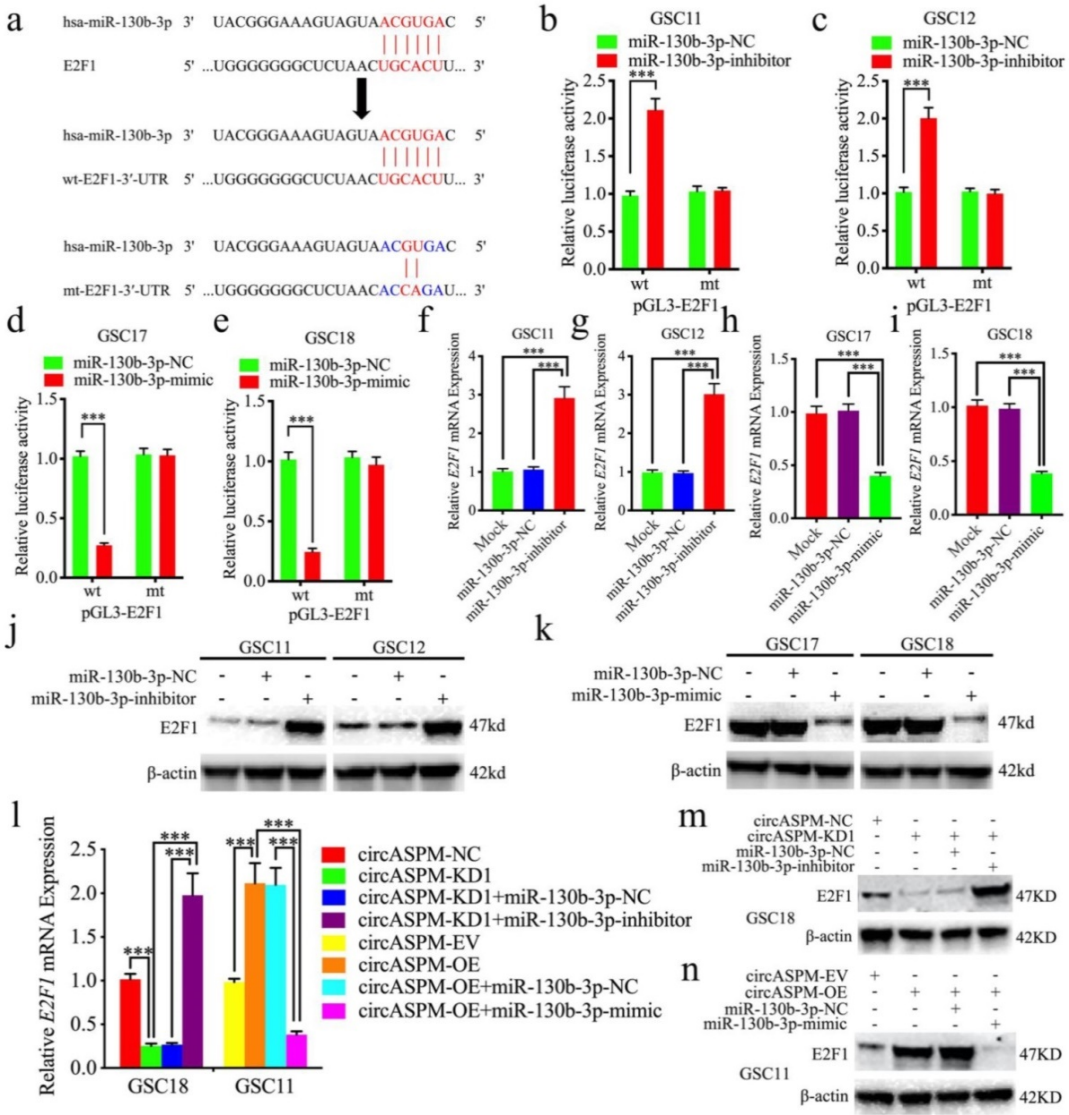
** CircAPSM regulate E2F1 expression via miR-130b-3p sponging.** a: Schematic diagram of the putative miR-130b-3p binding site in the 3′-UTR of E2F1. b, c, d, e: The luciferase reporter assays showed that miR-130b-3p mimic or inhibitor altered the luciferase promoter activities of E2F1. f, g, h, i, j, k: qPCR and western blotting showed the expression of E2F1 in GSCs after miR-130b-3p mimic or inhibitor treatment. l, m, n: The decreased or increased expression of E2F1 in GSCs induced by cARF1 knockdown or overexpression was reversed by miR-130b-3p inhibitor or mimic treatment, as determined by western blotting and qPCR. All data are shown as the mean ± SD (three independent experiments). *P < 0.05; **P < 0.01; ***P < 0.001.

Next, we intend to search for downstream targets of miR-130b-3p to determine the mechanism by which circAPSM exerts biological function. We searched StarBase 3.0 and found that miR-130b-3p can bind to the 3′-UTR of E2F1 (Fig. 5a). We detected the expression of E2F1 in glioma tissues and GSCs. The results showed E2F1 expressed highest in both glioma tissues and GSCs than NBT and NHA resprespectively (Sup. Fig. 3 b, d-g). Luciferase reporter assays showed that miR-130b-3p inhibitor treatment triggered an approximate doubling of the luciferase activity of E2F1-wt compared to the E2F1-mt group. In contrast, the luciferase activity of E2F1-wt was significantly decreased after miR-130b-3p mimic treatment (Fig. 5b-e). Both qPCR and western blotting showed a significant increase in E2F1 expression after miR-130b-3p inhibitor treatment in GSC11 and GSC12, and a decrease in E2F1 expression after miR-130b-3p mimic treatment in GSC17 and GSC18 (Fig. 5f-k). Finally, we again performed qPCR and western blotting to explore the effect of circASPM on E2F1 expression via miR-130b-3p sponging, and the results demonstrated that knockdown and overexpression treatment of circASPM in GSC18 and GSC11 could positively regulate the expression of E2F1. Meanwhile, the expression level of E2F1 was significantly reversed after rescue experiments with miR-130b-3p inhibitor or mimic treatment, respectively (Fig. 5l,m,n). Besides, the similar results were also obtained in glioma cell line U87 (Sup. Fig. 2 i, j). The above results prove that circAPSM regulates E2F1 expression through miR-130b-3p sponging.

### CircAPSM mediated GSCs proliferation via E2F1 expression

**Figure 6:**
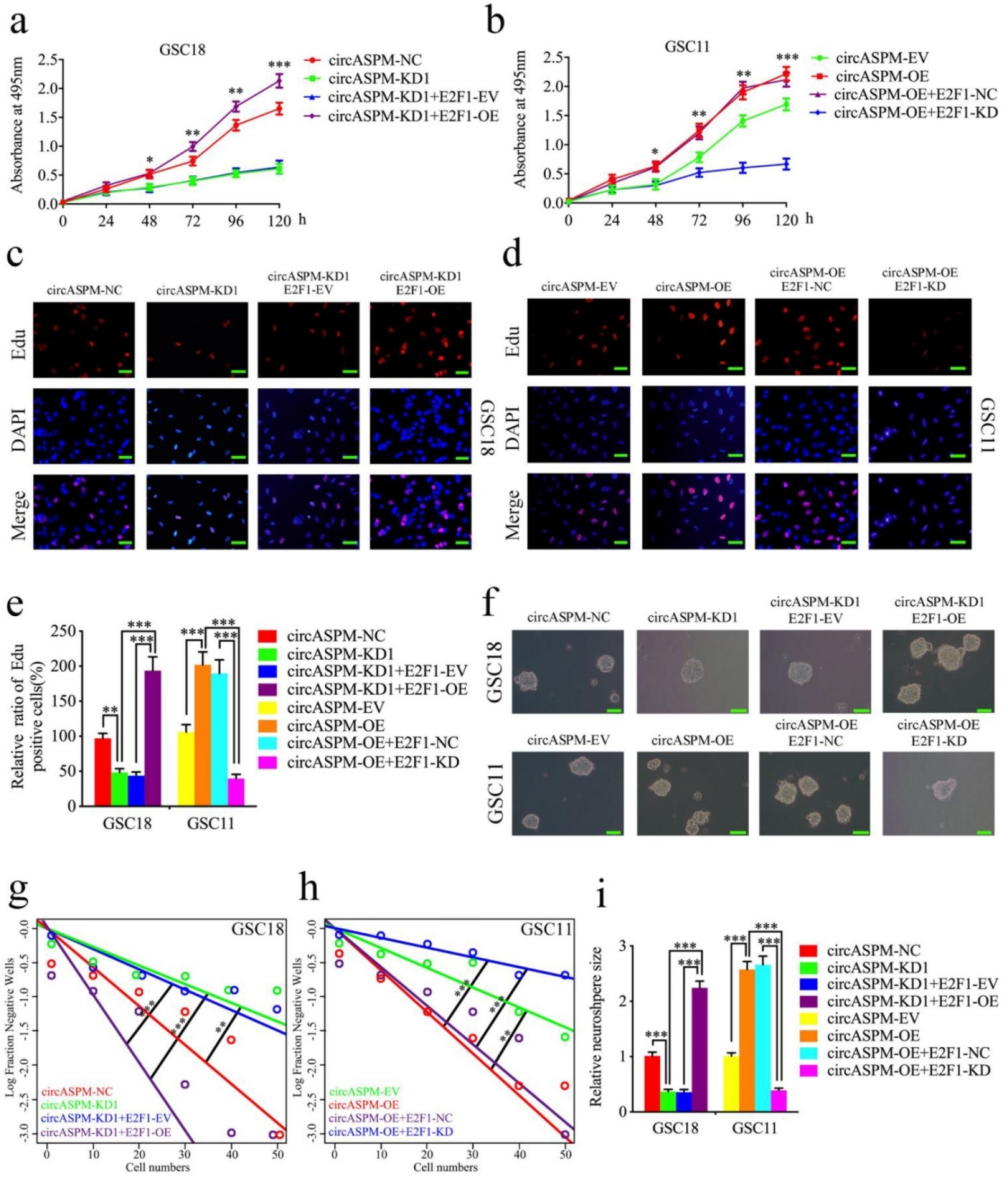
** CircAPSM mediated GBM cells proliferation via E2F1expression.** a,b: MTS assays showed that the GSCs viability regulated by circASPM knockdown or overexpression treatment were reversed by E2F1 overexpression or knockdown, respectively. c, d, e: The EDU assay showed that the GSCs proliferation regulated by circASPM knockdown or overexpression treatment were reversed by E2F1 overexpression or knockdown, respectively. Scale bar = 50 μm. f, g, h, i: The neurosphere formation assays and extreme limiting dilution assays showed that the circASPM transfection of overexpression plasmids or si-circASPM affected neurosphere growth capacity in GSCs and was reversed by miR-130b-3p E2F1 mimic or inhibitor treatment, respectively. Scale bar = 50 μm. All data are shown as the mean ± SD (three independent experiments). *P < 0.05; **P < 0.01; ***P < 0.001.

Since circASPM acts as a sponge of miR-130b-3p to regulate E2F1 expression, we further designed a series of experiments to determine whether circASPM promote the malignant phenotype of GBM via E2F1. MTS assays (Fig. 6a, b), EDU assays (Fig. 6c-e), neurosphere formation assays (Fig. 6f, i) and extreme limiting dilution assays (Fig. 6g, h) demonstrated that circASPM knockdown in GSC18 significantly decreased the viability, proliferation and formation rates of neurospheres, while these functions were all reversed after E2F1 overexpression. On the contrary, circASPM overexpression treatment of GSC11 up-regulated GSCs' viability, proliferation, and neurospheres formation rate, while the opposite results were also obatained after E2F1 knockdown. In addtiton, we performed the MTS assays in glioma cell line U87 and get the similar results (Sup. Fig. 2 k, l). Taken together, these results strongly suggest that circASPM promotes the malignant phenotype of GBM via miR-130b-3p mediated E2F1 regulating in GSCs.

### CircAPSM promoted GSCs tumorigenesis *in vivo*

**Figure 7:**
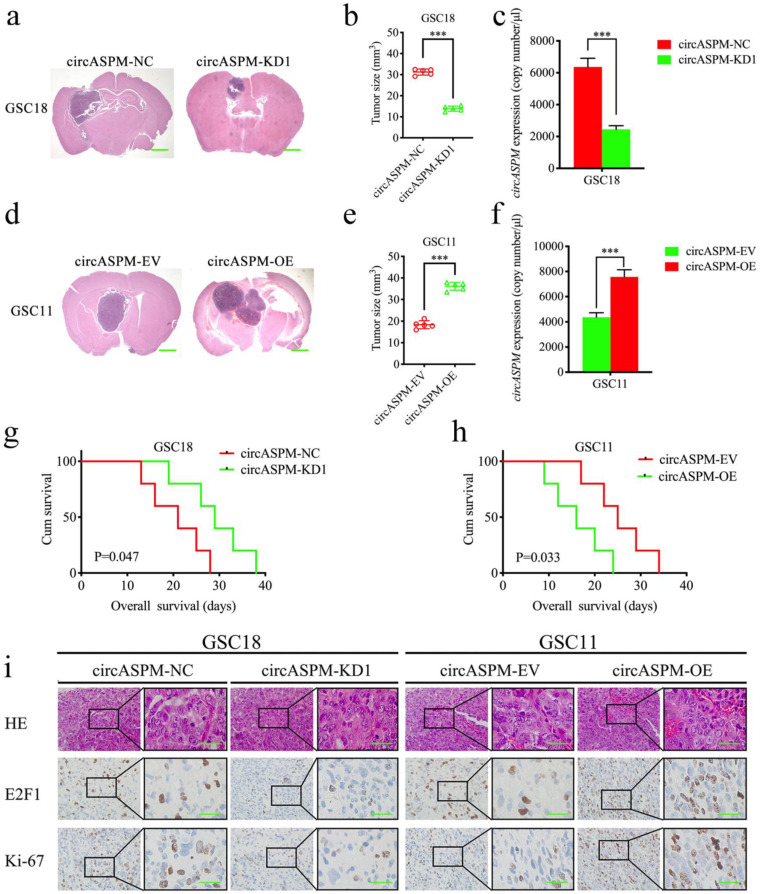
** CircAPSM mediated GBM cells proliferation via E2F1expression**. a, b: H&E-stained brain sections of mice with circAPSM knockdown in GSC18 were transplanted intracranially. The brain was harvested on the 15th day after transplantation. CircAPSM knockdown significantly inhibits tumor growth *in vivo*. Scale bar = 1 mm. c, f: The copy number of circASPM in tumor tissues derived from mice. d, e: H&E-stained brain sections of mice with circAPSM overexpression in GSC11 were transplanted intracranially. The brain was harvested on the 15th day after transplantation. CircAPSM overexpression significantly enhances tumor growth *in vivo*. Scale bar = 1 mm. g: Kaplan-Meier survival curve of circAPSM knockdown in GSC18 mice. h: Kaplan-Meier survival curve of circAPSM overexpression in GSC11. i: Representative immunohistochemical staining showed the changes in H&E, E2F1, and ki-67 staining in circAPSM overexpression and knockdown orthotopic xenograft models. Scale bar=50 μm. All data are shown as the mean ± SD (three independent experiments). *P < 0.05; **P < 0.01; ***P < 0.001.

We explored the role of circAPSM *in vivo* by establishing a tumor xenograft model. We found that knockdown of circAPSM resulted in a significant reduction in GSC18 tumor size (Fig. 7a, b) and a significantly longer median survival time (mean survival: 20.6±6.19 and 29±7.18 days; Fig. 7g). Comparatively, overexpression of circAPSM significantly promoted intracranial tumor growth in GSC11 (Fig. 7d, e) and reduced survival time (mean survival: 25.4±6.50 days, 16.2±6.02 days; Fig. 7h) compared to controls. In addition, absolute qPCR assays have confirmed the copy number of circASPM in the nude mice bearing tumor tissues (Fig. 7c, f). Through H&E staining and immunohistochemical staining, we found that the staining intensities and expression levels of circAPSM, E2F1 and ki-67 were significantly higher in the circAPSM overexpression group than in the control group, while we obtained opposite results in the knockdown group (Fig. 7i). The above results demonstrated that circAPSM promoted GSCs tumorigenesis *in vivo*.

## Discussion

The World Health Organization (WHO) classifies gliomas into four grades according to tumor cell morphology, including WHO grade I, II, III, IV. The most malignant grade IV glioma is also known as GBM 44, and more than half of all gliomas are GBM 1. The current traditional GBM treatment plan of surgical resection combined with radiotherapy and chemotherapy has a poor prognosis, such as a high postoperative recurrence rate and short survival time 45, 46. Therefore, an in-depth study of the course of GBM is imperative. Many recent studies have found that circRNAs can exert their biological effects through multiple pathways and pay contributions to the course of GBM 12-14. This study aims to validate the tumorigenic role of circASPM in GBM and detect the mechanisms by which it exerts its functions.

GEO analysis revealed that circASPM was expressed at higher levels in GBM compared to normal tissue and low-grade gliomas and that its expression level was positively correlated with patient survival time. We then cultured patient-derived GSCs cell line and used RNase R assays to prove that circASPM was stable after verifying that the cell lines culture worked well 42. Subsequently, we performed gain-of-function assays and loss-of-function assays to analyze the biological role of circASPM in GBM progression. We found that the expression level of circASPM can positively regulate the proliferation and growth of tumor cells, suggesting that circASPM plays an important regulatory role in the development of GBM.

Many researchers have found very complex links and interactions between different types of RNA 10. CircRNA can act as a ceRNA to regulate target genes' expression through miRNA sponging 11, 47. We found a binding site for miR-130b-3p on circAPSM by searching StarBase 3.0 in our study, and subsequent assays also proved our prediction. According to our data, the expression of miR-130b-3p was inversely correlated with circASPM *in vivo*. In addition, miR-130b-3p inhibitor treatment and mimic treatment could eliminate the promoting or inhibiting effects of circASPM overexpression or knockdown, respectively.

It has long been reported that miRNAs can bind to the 3′-UTR of mRNA to perform their own biological functions 48. So we further probed and found that E2F1 is a direct target of miR-130b-3p. E2F1 mediates regulation of the cell cycle, and its aberrant activation is strongly associated with poor prognosis in a variety of cancers. E2F1 pays great contributions to promote the development and maintains the stemness of tumor cells in gastric cancer, prostate cancer or some other tumors 49, 50. In glioma, E2F1 can be used as a target for miR-205, miR-371 to promote glioma growth and invasion 51, 52. Our results showed that both mRNA and protein levels of E2F1 were up-regulated in GSCs tissues and cells in response to circASPM overexpression, exerting stronger tumor-promoting effects. Subsequent rescue experiments of E2F1 reversed the biological effects produced by circASPM overexpression or knockdown, corroborating that circASPM regulates E2F1 expression by acting as a ceRNA for miR-130b-3p.

Finally, we performed xenograft experiments and found that overexpression of circASPM significantly promoted the growth of intracranial tumors and shortened the survival time of mice. The staining intensity and expression levels of circAPSM, E2F1, and ki-67 were significantly higher than those in the control group, whereas in the knockdown group, we obtained the opposite results. These data mentioned above prove that circAPSM promotes GSCs tumorigenesis *in vivo*. However, this study lacks depth in exploring more specific mechanisms such as angiogenesis, signaling pathways, etc., and warrants continued follow-up studies.

## Conclusion

In conclusion, this study demonstrates that circASPM is highly expressed in GBM, which up-regulates the expression level of E2F1 by acting as a ceRNA for miR-130b-3p, promotes a range of malignant behaviors such as growth, proliferation and invasion, and leads to a poor patient prognosis. Subsequent studies and clinical translation against circASPM would be valuable.

## Supplementary Material

Supplementary figures and tables 1-2.

Supplementary table 3: The expression difference of ASPM derived circRNAs in GSE109569.

Supplementary table 4: ENCORI_hg19_CLIP-seq_miRNA-target_all_ASPM.

## Abbreviations

CircRNAs: Circular RNAs
ncRNAs: non-coding RNAs
ceRNAs: endogenous RNAs
miRNAs: microRNAs
WHO: World Health Organization
GBM: Glioblastoma multiforme
GSCs: Glioma stem cells
MREs: microRNA response elements
GEO: Gene Expression Omnibus
H&E stain: hematoxylin and eosin stain
IHC: Immunohistochemistry
qRT-PCR/qPCR: Real-Time Quantitative Reverse Transcription PCR
RISC: RNA-induced silencing complex
