# Supplementary material for: Circular RNA circASPM promotes the progression of glioblastoma by acting as a competing endogenous RNA to regulate miR-130b-3p/E2F1 axis
Source: J Cancer. 2022 Mar 14;13(5):1664–78. doi: 10.7150/jca.57691 (PMC8965134; doi:10.7150/jca.57691)
Supplement: Supplementary file 1 — Supplementary figures and tables 1-2. [file jcav13p1664s1.pdf]

Supplementary figure 1

Isolation and validation of patient derived glioma stem cells.

a: H&E-stained images of the original patient tumors of GSC11, GSC12, GSC14, GSC16, GSC17 and GSC18.

b: GSCs adhered and differentiated into GFAP- or β-III tubulin-positive cells. Scale bar = 50 μm.

c: Neurospheres composed of $CD133^+$ / $Nestin^+$ GSCs were isolated from the primary culture. Scale bar = 50 μm.

d: PLOD1 mRNA expression in patient-derived GSCs as measured by qPCR.

e, f: qPCR assays detected the expression level of ASPM founding that overexpression and knockdown of circASPM did not affect the RNA expression level of ASPM.

All data are shown as the mean ± SD (three independent experiments). $*P < 0.05$; $**P < 0.01$; $***P < 0.001$.

Supplementary figure 2

circASPM promotes the proliferation of U87 via miR-130b–3p/E2F1 axis.

a: The expression of circASPM in U87 after transfection of the circASPM overexpression plasmids as measured by qPCR.

b: circASPM overexpression significantly increased the proliferation of U87 in MTS assays.

c, d : Proliferative capacity of tumor cells was inhibited after circASPM overexpression in U87 as measured by Edu assays. Scale bar = 50μm.

e, f: The expression of miR-130b-3p was down-regulated after circAPSM overexpression while circASPM knockdown up-regulated miR-130b-3p expression as measured by qPCR in U87.

g: The luciferase reporter assays showed that miR-130b-3p inhibitor altered the luciferase promoter activities of circASPM in U87.

h: The luciferase reporter assays showed that miR-130b-3p inhibitor altered the luciferase promoter activities of E2F1.

i, j: qPCR and western blot showed the expression of E2F1 in GSCs after miR-130b-3p inhibitor treatment.

k: MTS assays showed that circASPM transfection of overexpression plasmids affected GSCs viability and was reversed by miR-130b-3p mimic treatment.

l: MTS assays showed that the GSCs viability regulated by circASPM overexpression treatment were reversed by E2F1 knockdown in U87.

All data are shown as the mean ± SD (three independent experiments). *P < 0.05; **P < 0.01; ***P < 0.001.

Supplementary figure 3

The expression levels of miR-130b-3p and E2F1 in glioma tissues and cells

a: MiR-130b-3p expression was correlated with WHO grades in glioma.

b: E2F1 expression was negatively correlated with WHO grades in glioma.

c: Mir-130b-3p mRNA expression in patient-derived GSCs as measured by qPCR.

d: E2F1 mRNA expression in patient-derived GSCs as measured by qPCR.

e, f: E2F1 protein expression in glioma tissues as measured by western blotting (e) and immunohistochemistry (f).

g: E2F1 protein expression in patient-derived GSCs as measured by western blotting.

All data are shown as the mean ± SD (three independent experiments). *P < 0.05; **P < 0.01; ***P < 0.001.

Supplementary figure 4

a: The difference expression of circASPM between human glioma tissues and mice xenograft tumor tissues (GSC11 and GSC18).

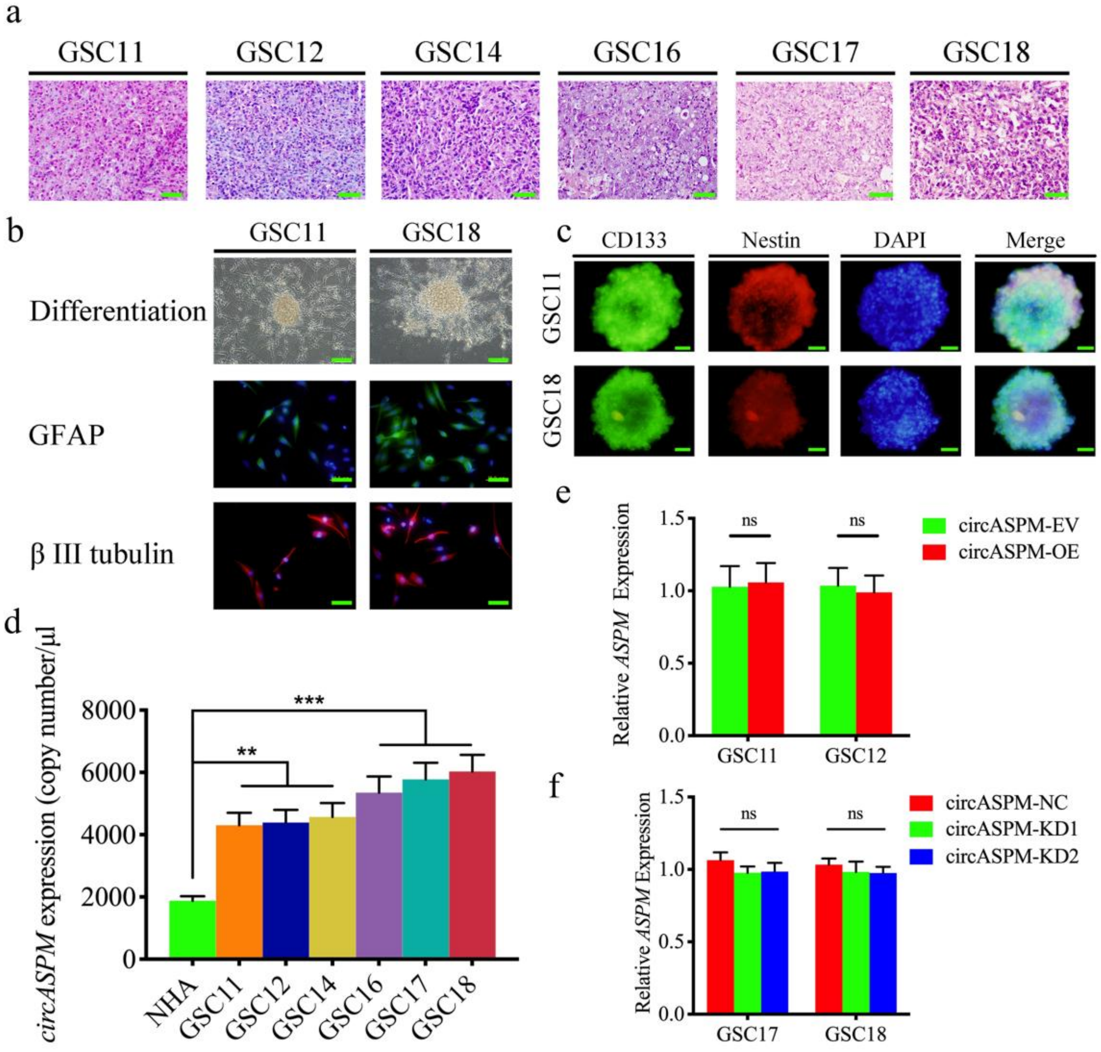

a
GSC11
GSC12
GSC14
GSC16
GSC17
GSC18
b
GSC11
GSC18
Differentiation
GFAP
β III tubulin
c
CD133
Nestin
DAPI
Merge
GSC11
GSC18
d
circASPM expression (copy number/μl
8000
6000
4000
2000
0
***
**
NHA
GSC11
GSC12
GSC14
GSC16
GSC17
GSC18
e
Relative ASPM Expression
1.5
1.0
0.5
0.0
ns
ns
GSC11
GSC12
circASPM-EV
circASPM-OE
f
Relative ASPM Expression
1.5
1.0
0.5
0.0
ns
ns
GSC17
GSC18
circASPM-NC
circASPM-KD1
circASPM-KD2

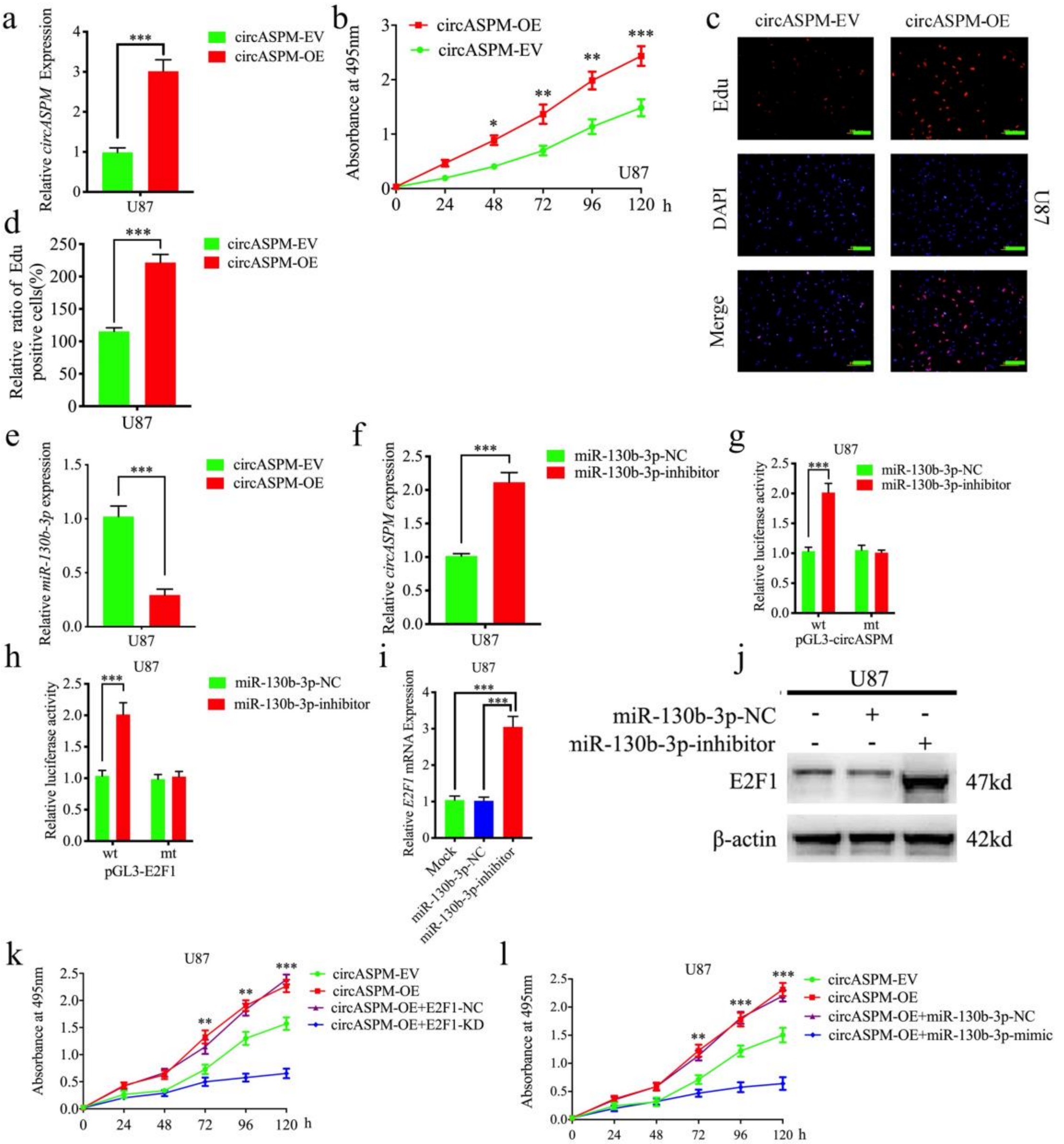
a
Relative circASPM Expression
circASPM-EV
circASPM-OE
U87
b
Absorbance at 495nm
circASPM-OE
circASPM-EV
U87
h
c
circASPM-EV
circASPM-OE
Edu
DAPI
Merge
U87
d
Relative ratio of Edu positive cells(%)
circASPM-EV
circASPM-OE
U87
e
Relative miR-130b-3p expression
circASPM-EV
circASPM-OE
U87
f
Relative circASPM expression
miR-130b-3p-NC
miR-130b-3p-inhibitor
U87
g
U87
Relative luciferase activity
miR-130b-3p-NC
miR-130b-3p-inhibitor
wt
mt
pGL3-circASPM
h
U87
Relative luciferase activity
miR-130b-3p-NC
miR-130b-3p-inhibitor
wt
mt
pGL3-E2F1
i
U87
Relative E2F1 mRNA Expression
Mock
miR-130b-3p-NC
miR-130b-3p-inhibitor
j
U87
miR-130b-3p-NC
miR-130b-3p-inhibitor
E2F1
47kd
β-actin
42kd
k
U87
Absorbance at 495nm
circASPM-EV
circASPM-OE
circASPM-OE+E2F1-NC
circASPM-OE+E2F1-KD
h
l
U87
Absorbance at 495nm
circASPM-EV
circASPM-OE
circASPM-OE+miR-130b-3p-NC
circASPM-OE+miR-130b-3p-mimic
h

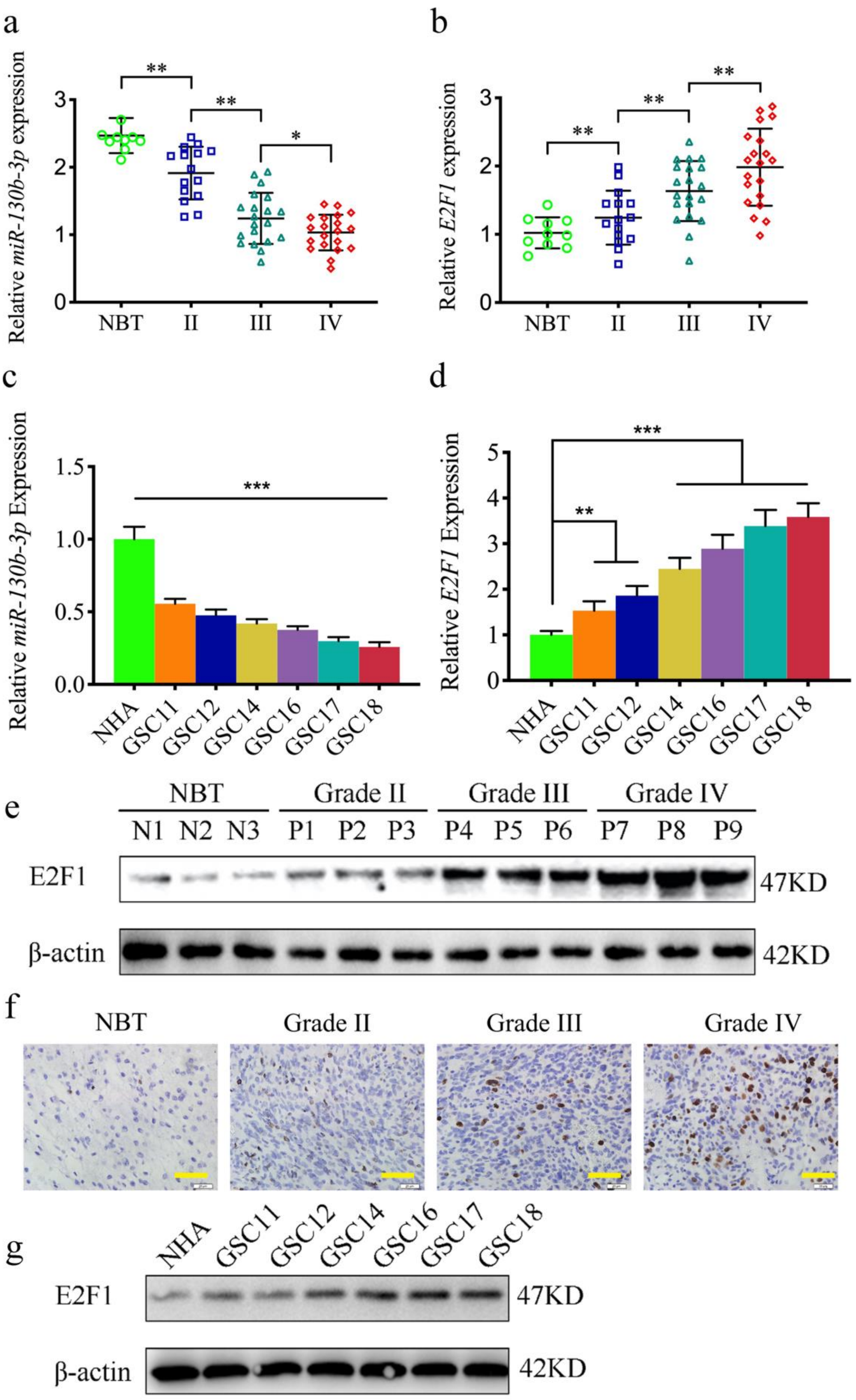

a
Relative miR-130b-3p expression
NBT
II
III
IV
b
Relative E2F1 expression
NBT
II
III
IV
c
Relative miR-130b-3p Expression
NHA
GSC11
GSC12
GSC14
GSC16
GSC17
GSC18
d
Relative E2F1 Expression
NHA
GSC11
GSC12
GSC14
GSC16
GSC17
GSC18
e
NBT
Grade II
Grade III
Grade IV
N1 N2 N3 P1 P2 P3 P4 P5 P6 P7 P8 P9
E2F1
47KD
β-actin
42KD
f
NBT
Grade II
Grade III
Grade IV
g
NHA
GSC11
GSC12
GSC14
GSC16
GSC17
GSC18
E2F1
47KD
β-actin
42KD

a

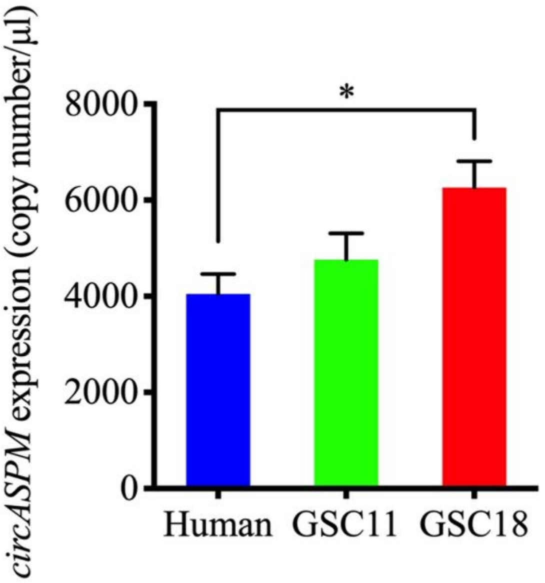

circASPM expression (copy number/μl)
8000
6000
4000
2000
0
*
Human
GSC11
GSC18

Table S1. Relationship of circASPM expression to clinical features of glioma patients.

**Clinical features**		**Samples (*n* = 55)**	**circASPM expression**		***P* value**
			**Low (*n* = 24)**	**High (*n* = 31)**	
Sex	Male	27	11	16	0.6707
	Female	28	13	15	
Age	≤ 50	30	11	19	0. 2536
	> 50	25	13	12	
WHO grade	II	15	11	4	**0.0159**
	III	20	8	12	
	IV	20	5	15	

CircASPM expression was detected by immunohistochemistry and evaluated according to the German immunohistochemical score. High expression was defined as score ≥4.

Supplementary Table 2. Clinical information of the primary glioma stem-like cells

	GSC11	GSC12	GSC14	GSC16	GSC17	GSC18
Gender	Male	Female	Female	Female	Male	Male
Age	66 years old	68 years old	53 years old	36 years old	58 years old	37 years old
Location	Left parietal lobe	Right insula	Right parietal lobe	Left occipital lobe	Right insula	Left parietal lobe
WHO grade	IV	IV	IV	IV	IV	IV
Ki-67	40% (+)	40% (+)	60% (+)	60% (+)	70% (+)	60% (+)
